# Behavior of neuropathic pain in mice following chronic constriction injury comparing silk and catgut ligatures

**DOI:** 10.1186/s40064-015-1009-4

**Published:** 2015-05-15

**Authors:** Selina van der Wal, Lisa Cornelissen, Marije Behet, Michiel Vaneker, Monique Steegers, Kris Vissers

**Affiliations:** Department of Anesthesiology, Pain and Palliative Medicine, Radboud University Nijmegen Medical Centre (RUNMC), 6525 GA, Nijmegen, the Netherlands; Department of Microbiology RUNMC, 6525 GA, Nijmegen, the Netherlands

**Keywords:** Allodynia, Catgut, Hyperalgesia, Neuropathic pain, Silk

## Abstract

**Introduction:**

Neuropathic pain is defined as pain arising as a direct consequence of a lesion or disease affecting the somatosensory system and is common after surgery. Neuropathic pain can persist without an obvious injury. In this study we aim to validate a murine chronic constriction injury model as a model for neuropathic pain research and determine if silk or catgut ligatures induced most stable neuropathic pain behavior.

**Methods:**

In this study mice underwent chronic constriction or sham surgery. Mice were tested on cutaneous hyperalgesia with the cumulative reaction time in the acetone test, on allodynia with the cumulative reaction time and number of lifts in the cold plate test and the maximal force before withdrawal in von Frey test.

**Results:**

In the acetone test neuropathic pain was seen in CCI mice, but not in sham mice. Hyperalgesia was present postoperatively in CCI mice compared with preoperatively. In the cold plate test cumulative reaction time and number of lifts were higher in the ipsilateral hind paw than in the contralateral hind paw and sham mice. Postoperative measurements were higher than preoperatively. In the von Frey test the postoperative measurements were lower in the ipsilateral hind paw than preoperatively, while the contralateral hind paw showed an increase in maximal force before withdrawal. The contralateral hind paw showed more difference with sham mice than the ipsilateral hind paw. Silk ligatures showed more stable neuropathic pain behavior. In the acetone test, the cold plate test and the von Frey test the mice scored higher on neuropathic pain having silk ligatures, compared with catgut ligatures.

**Conclusion:**

In this study we validated a murine CCI model for neuropathic pain behavior. In the murine CCI model it appears that silk ligatures demonstrate more stable neuropathic pain behaviors than catgut ligatures in de CCI model.

**Electronic supplementary material:**

The online version of this article (doi:10.1186/s40064-015-1009-4) contains supplementary material, which is available to authorized users.

## Introduction

Neuropathic pain is defined as pain arising as a consequence of a lesion or disease affecting the somatosensory system and is common after surgery (Treede et al. 2008). Neuropathic pain presents as a constant, burning pain with spontaneous sharp exacerbations and worsening upon normal sensory triggers (Dieleman et al. 2008).

The grading system of neuropathic pain is based on certain criteria, explained by Treede (Treede et al. 2008). The criteria consist of the distribution of pain coupled to the medical history and clinical investigation with supplemental sensory testing. Depending on the number of criteria that match with the patient, neuropathic pain is confirmed or excluded (Treede et al. 2008). Sensory testing is an important diagnostic tool in determination of neuropathic pain and allodynia and hyperalgesia should be determined (Macrae 2001).

Neuropathic pain is associated with poor physical and mental health and adversely affects quality of life (Freynhagen & Bennett 2009; Leung & CM Cahill 2010). The prevalence of neuropathic pain in the human population ranges from 1 to 17.9% (Hecke van, O et al. 2013). considering the above neuropathic pain adds to the burden of direct and indirect medical cost for our society (Leung & C.M. Cahill 2010), as there are direct medical costs, loss of the ability to work, loss of caregivers' ability to work and possibly greater need for institutionalization or other living assistance (O'Connor 2009).

The treatment of neuropathic pain mostly consists of oral analgesics such as tricyclic anti-depressants (TCAs) and anti-epileptic drugs (AEDs) (Allen 2005), to decrease the symptoms of neuropathic pain. However, the therapeutic response on the pharmacological treatment of neuropathic pain is rather poor, as few patients receive efficacious dosages of medication (Dieleman et al. 2008; O'Connor 2009).

Because the mechanisms of neuropathic pain are insufficiently understood, (Clark et al. 2013; Thacker et al. 2007) it seems pivotal to investigate the course and cause of neuropathic pain and development of treatment and perhaps prevention strategies. Therefore ideally we want to study a neuropathic pain animal model extrapolatable to the clinical situation. Often a chronic constrictive injury model is used in rats to study neuropathic pain which include thermal and mechanical allodynia testing (Bennett & Xie 1988; Mogil 2009). In this study we aim to validate a chronic constriction model in mice. A murine model can lead to a better understanding of the course of neuropathic pain, and will lead to an improvement of accuracy and variability of the chronic constriction model, because of the possibility to use transgenic mice (Mogil 2009).

The material that is used for ligatures can have an effect on the outcome of the observed sensory abnormalities (Robinson & Meert 2005). In chronic constriction injury, either catgut or silk ligatures are used. In rats, catgut is commonly used as ligature material (Gabay & Tal 2004). Catgut leads to a development of an inflammatory reaction and consequentially a loss of most A-fibres and some C-fibers, but few cell bodies (Bridges et al. 2001; Selvig et al. 1998). In mice, however, the preferable ligature material is not known. In this research both silk and catgut ligatures were compared, to check for efficacy in inducing neuropathic pain behavior.

In this study we aim to validate a model of neuropathic pain in mice and investigate whether silk or catgut ligature material is more effective in inducing neuropathic pain.

## Methods

All experiments were approved by the Regional Animal Ethics Committee in Nijmegen and performed under the guidelines of the Dutch Council for Animal Care and The National Institutes of Health.

### Study population

All studies were performed in C57BL/6J male mice (Charles River). Mice were aged 6 weeks upon arrival were first acclimatized. Mice were housed in a light and temperature controlled room under specific pathogen free (SPF) conditions. Standard pelleted chow (1.00% Ca; 0.22% Mg; 0.24% Na; 0.70% P; 1.02% K; SSNIFF Spezialdiäten GmbH, Soest, Germany) and drinking water were available *ad libitum*.

### Experimental design

This experiment was used to validate the chronic constriction injury (CCI) model in mice (n=45) to induce neuropathic pain. In the experiment postoperative testing was done in both sham-group (n=5) and CCI-group (n=40), with either catgut (n=20) or silk (n=20) ligatures.

### Surgical procedure

Both sham and CCI-mice were being operated. Before surgery, the mice got rimadyl subcutaneously according to their weight (0.1 ml rimadyl per 10 gram). The mice were anesthetized using isofluran inhalation (1–4%). Under a dissecting microscope, the left common sciatic nerve was exposed at the level of the mid-thigh by dissecting through the biceps femoris. In contrary to the sham-mice, in which no ligatures were placed, in de CCI-mice, proximal to the nerve trifurcation (while taking care to preserve epineural circulation), three ligatures (either silk 6.0 or catgut 6.0) were loosely tied around the sciatic nerve, at about 1 mm spacing, until they elicited a brief twitch in the related hind paw. The muscle layer was then stitched and the incision in the shaved skin layer was closed using suture or clips. The sham-operated animals were used as controls and had only sciatic exposure without ligation. Also after surgery the mice got rimadyl subcutaneously according to their weight on day 1 and day 2 once a day. On day 10, the clips were removed. After the experiment, when the mice were euthanized, the nerve histology was studied by removing connective tissue and ligatures.

### General well-being

The first week after surgery, animals will be weighted daily. When the animal loses too much weight (>30% directly after surgery or 20% not directly after surgery, starting weight mean of approximately 24 grams) or does not recover within 1 week the humane endpoint has been reached and the animal will be excluded from the experiment and consequently postoperative pain testing. They were also tested on activity, state of the surgical wound and eventual damage on the left feet or toes (by autotomy).

### Postoperative testing

Responses to thermal and mechanical stimuli were tested in all mice. Sham mice were tested before surgery (baseline) and 3, 7, 10, 14 and 21 days after surgery. CCI-mice were tested before surgery (baseline) and 3, 7, 10, 14, 21 and also 28 days after surgery

Thermal and chemical hyperalgesia were tested using the acetone spray test. After habituation for at least 15 minutes in plexiglass cubicles with a wire mesh metal floor, the plantar area of the left hind paw was exposed to acetone. For one minute the mouse was scored on lifting up the paw, scratching to the paw and touching the left hip or paw. The duration of the reaction was measured and analysed as cumulative reaction time (Vissers & Meert 2005).

Thermal allodynia was measured using the Cold Plate test. The mice were exposed to a temperature of 2–2.5°C to regain the best response. Measurements were performed on both the ipsilateral and contralateral hind paws. Mice were scored for 5 minutes on scratching with a paw, lifting up the paw, lifting up the paw shortly in the same place and licking on the toes. The amount of lifting of the hind paw was measured and analysed as number of lifts. Also the duration of reaction was measured, analysed as cumulative reaction time (Bennett & Xie 1988; Jasmin et al. 1998). However, the cold plate test became defective, so in some groups the number of mice with catgut ligatures is lower.

Mechanical allodynia was measured using the Von Frey test, preoperatively and on day 4, 7, 11, 14, 18, 21 and 27. Mechanical allodynia is induced by application of pressure to the skin (Field et al. 1999). The mice were placed in a test cage with a wire mesh metal floor and the rigid tip of a von Frey filament (punctate stimulus) was applied to the skin of the midplantar area of the hind paw until it bends. Different filaments, ranging from 0.145 to 5.1 gram (Table 1), made of nylon, were used that exerted an increasing force, starting below the threshold of detection (hair number 7 or 8; 0.145–0.320 gram) and increasing until the animal removed its paw. Withdrawal threshold of ipsilateral and contralateral paws was measured 3–5 times and the maximal force before withdrawal was the mean of the evaluations (Chaplan et al. 1994).

**Table 1:** **Number of the von Frey hair with the corresponding weight in grams**

**Table 1.**	
**Number of the hair**	**Weight of the hair (g)**
**4**	0.03
**6**	0.09
**7**	0.15
**8**	0.32
**9**	0.39
**10**	1.1
**11**	1.7
**12**	3.3
**13**	5.1
**14**	8.3

### Statistical analysis

Results are presented as mean values ± S.E. All statistical analyses were performed with IBM SPSS Statistics 20 (SPSS, Chicago, IL). Because of some missing data in the CCI group statistical analysis of post- and pre-measurements in the acetone test, cold plate test and the von Frey test were done using linear mixed models. For the analysis of ligature material and the differences between CCI and sham mice a multifactor ANOVA-test is performed. A p-value of 0.05 is considered statistically significant. To determine the experimental group size a calculation on data is performed based on previously published information (Osikowicz et al. 2008), using the following formula: n = 1 + 2C(s/d)2 (Dell et al. 2002) to compute sample size for continuous variables where s is an estimation of the standard deviation of the variable, d is the magnitude of the difference we wish to be detected, and C is a constant dependent on the value of alpha and beta selected. C = 10.5 for α = 0.5 and 1-β = 0.9, then sample size is n = 1 + 21 × (5/10)2 = 6.25. This analysis showed that to detect differences of 10% with a power of 90% and statistical significance at the p < 0.05 level, 7 mice per group is needed. Since we expect that 10–30% of animals that will undergo chronic construction injury will not have successful neuropathic symptoms 10 animals per group will be needed to produce statistically valid results.

## Results

### General well-being

There were no complications after surgery and no animals had to be excluded from the study. One mouse got a staphylococcus infection at the end of the experiment, which was treated. The fur of all the mice was clean, shining and well groomed.

Directly after surgery, CCI-mice showed a characteristic posture of the left hind paw, with a curve downward and decreased musculature and thereby made abnormal movements with the left hind paw. After approximately one week, the enlarged movements were still seen and the left hind paw was still curved downwards, but the other abnormal movements mostly disappeared. Some mice showed mild signs of autotomy, as they gnaw or bite their paws or toes, which could indicate that the ligation of the sciatic nerve was too tight. There were occasional signs of stress present, but no abnormal aggression amongst the mice was seen. The activity of the mice was generally normal. Both sham as CCI-mice gained weight during the experiment. After the experiment it was confirmed that constrictions were still present.

### Acetone test

**Figure 1:**
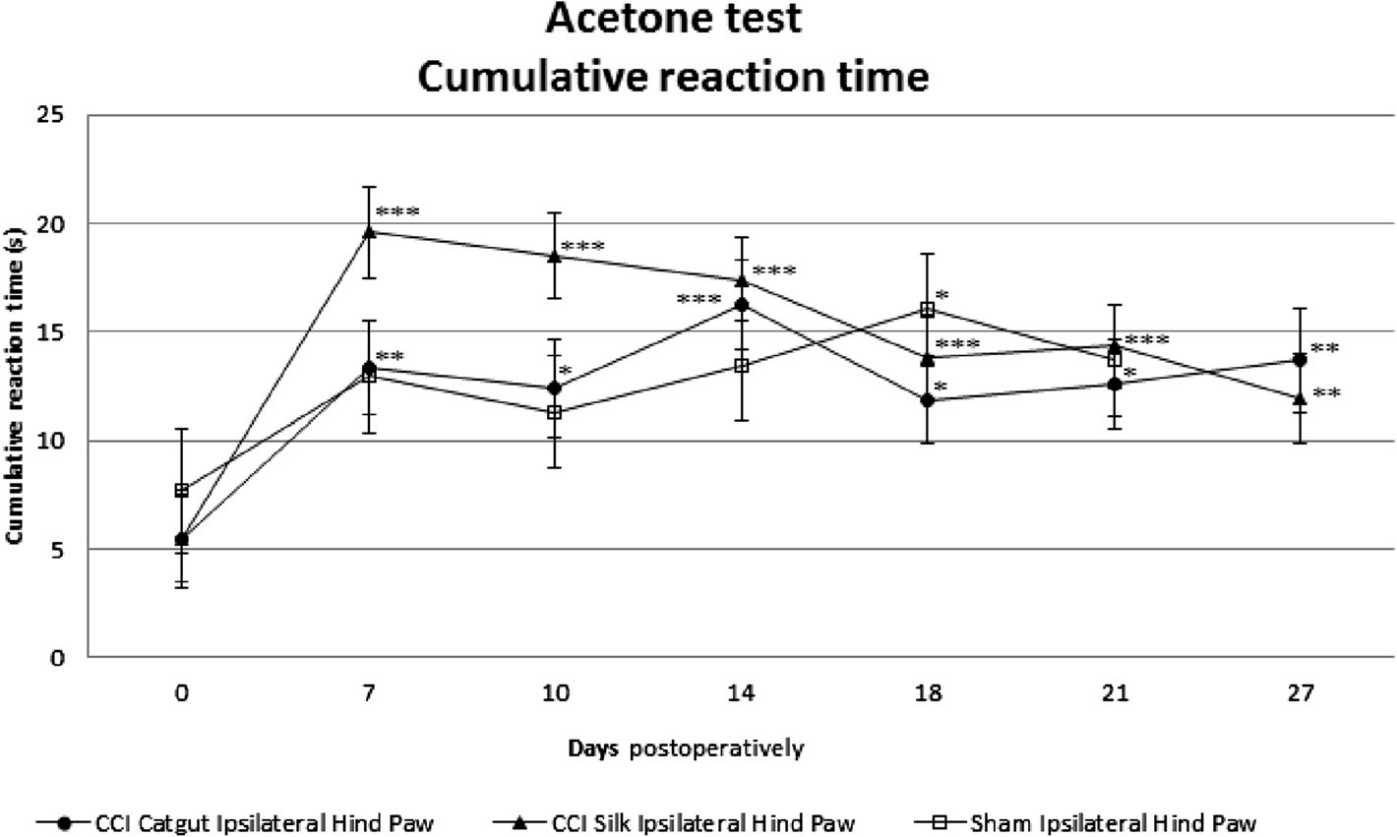
Thermal and chemical hyperalgesia (cumulative reaction time ±SE) measured in CCI mice and sham mice with acetone test preoperatively and postoperatively. Statistics were done with linear mixed models in which: * p<0.05 as compared with preoperative measurement, **p<0.01 as compared with preoperative measurement, ***p<0.001 as compared with preoperative measurement. Measurements on day 3 and day 33 were left out, because of missing data. A significant increase was seen in both CCI mice, silk and catgut ligatures, from day 0 until day 7. In silk a significant decrease is seen after day 7.

To test thermal hyperalgesia the acetone test was performed in sham and CCI-mice (Figure 1). According to the acetone test an increase in cumulative reaction time in sham mice was seen. This increase was significant postoperatively compared with preoperatively on day 18 and 21. In CCI mice a significant increase in cumulative reaction was seen preoperatively compared with day 7, in silk ligatured mice more than in catgut ligatured mice. From day 7 the cumulative reaction time decreased in the silk group, but was still higher than preoperatively, which indicates neuropathic pain following CCI in mice according to the acetone test. In the catgut ligatured mice the cumulative reaction time increased until day 14 but then showed a decline on day 18. All postoperative measurements in catgut ligatured group were significantly higher than preoperatively. However, there was never a significant difference between sham mice and CCI mice. Silk ligatures induce a higher postoperative respons to acetone than catgut ligatures (p=0.042).

### Cold plate test

**Figure 2:**
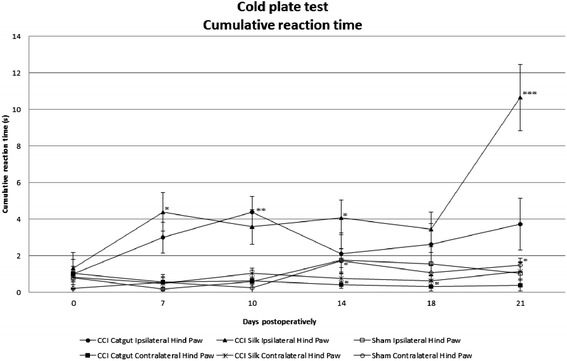
Thermal allodynia measured in CCI mice and sham mice with the cold plate test in cumulative reaction time ±SE preoperatively and postoperatively in both ipsilateral (left) and contralateral (right) hind paw. Statistics were done with linear mixed models in which: *p<0.05 as compared with preoperative measurement, **p<0.01 as compared with preoperative measurement, ***p<0.001 as compared with preoperative measurement. In CCI-mice with silk ligatures a significant increase is seen from preoperatively to day 10. From day 10 until day 14 a decrease in cumulative reaction time was seen, though this difference was not significant. In CCI-mice with silk ligatures a significant increase of cumulative reaction time was seen on day 7, day 14 and day 21 compared with the preoperative cumulative reaction time.

With the cold plate test cumulative reaction time as well as number of lifts is measured in left and right hind paw (Figure 2). Sham mice and the contralateral hind paw showed an equal cumulative reaction time, while cumulative reaction time increased in CCI mice, in silk ligatured mice more than catgut ligatured mice. The cumulative reaction time in the silk ligatured mice was still increasing on day 21, while catgut ligatured mice showed a decrease in cumulative reaction time on day 14. A difference in sham mice and the right hind paw is shown on day 14 (p=0.007) and day 18 (p=0.037). In comparison the ipsilateral hind paw and the sham mice were different on day 7, day 10, day 18 and day 21.

**Figure 3:**
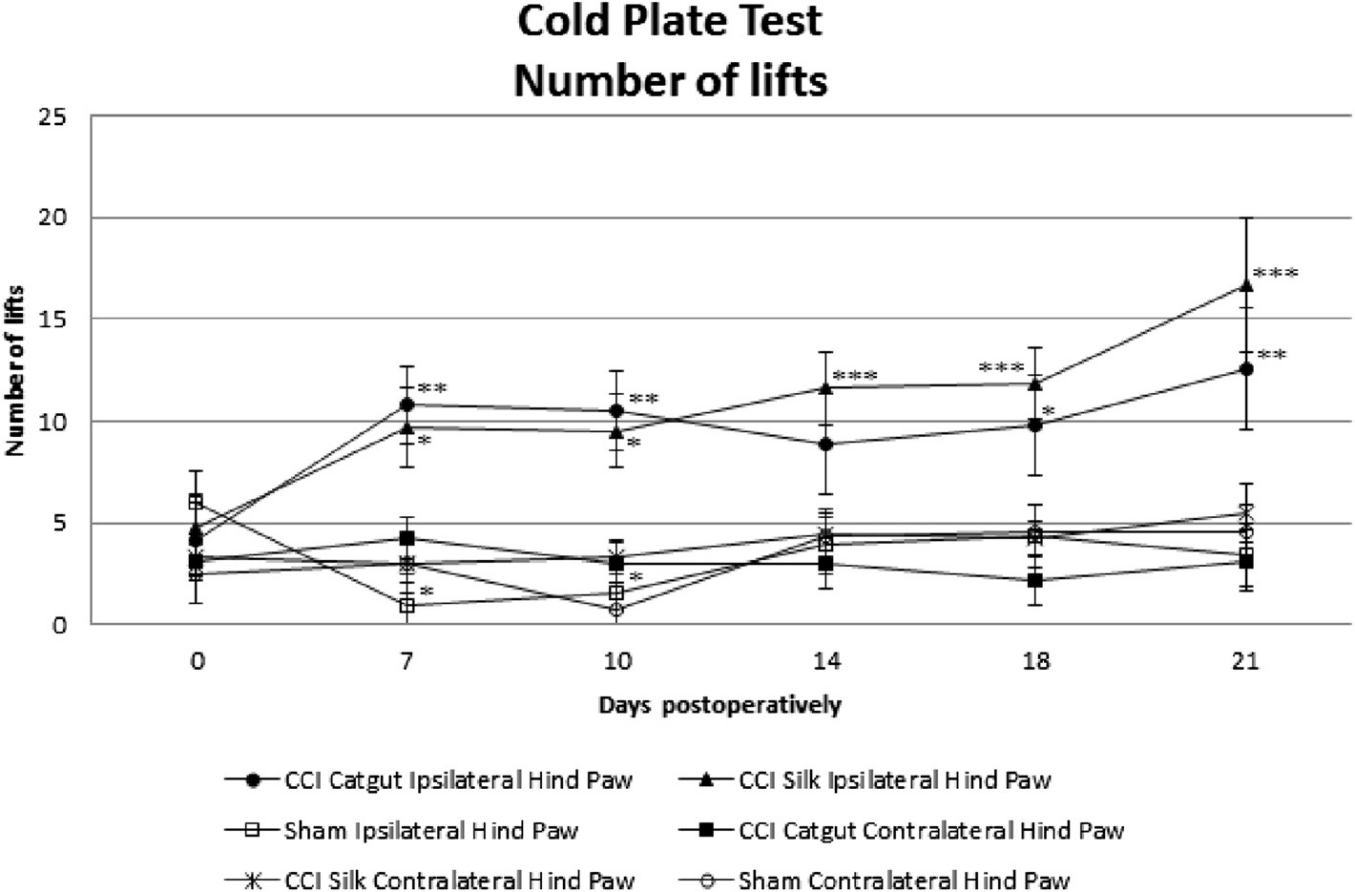
Thermal allodynia measured in CCI mice and sham mice with cold plate test in number of lifts ±SE preoperatively and postoperatively in both ipsilateral (left) and contralateral (right) hind paw. Statistics were done with linear mixed models in which: *p<0.05 as compared with preoperative measurement, **p<0.01 as compared with preoperative measurement, ***p<0.001 as compared with preoperative measurement. A significant increase was seen from preoperatively to day 7 in both silk and catgut ligatured mice considering the left hind paw. In CCI mice with silk ligatures also a significant increase was seen from day 18 to day 21. In the CCI mice considering the right hind paw and the sham-mice the number of lifts remained constant.

Thermal allodynia was also tested by the cold plate test considering the number of lifts (Figure 3). Sham mice and right hind paw remained constant over time. Sham mice even showed a significant decrease in number of lifts of day 7 and 10. The number of lifts in CCI mice significantly increased. The catgut ligatured CCI mice showed an increase in number of lifts, until a decrease in number of lifts was seen on day 14, on which the postoperative measurement was not significantly different to the preoperative measurement. In silk ligatured mice a constant increase in number of lifts was seen and the number of lifts was still increasing on day 21. Neuropathy in mice according to cumulative reaction time as well as number of lifts significantly increased from day 7 in the left hind paw of CCI mice and decreased from day 7 in the right hind paw of CCI mice. The number of lifts of sham mice and the contralateral hind paw differed from the ipsilateral hind paw in all postoperative measurements, but not in the preoperative measurement, which indicates neuropathic pain following the cold plate test.

### Von Frey test

**Figure 4:**
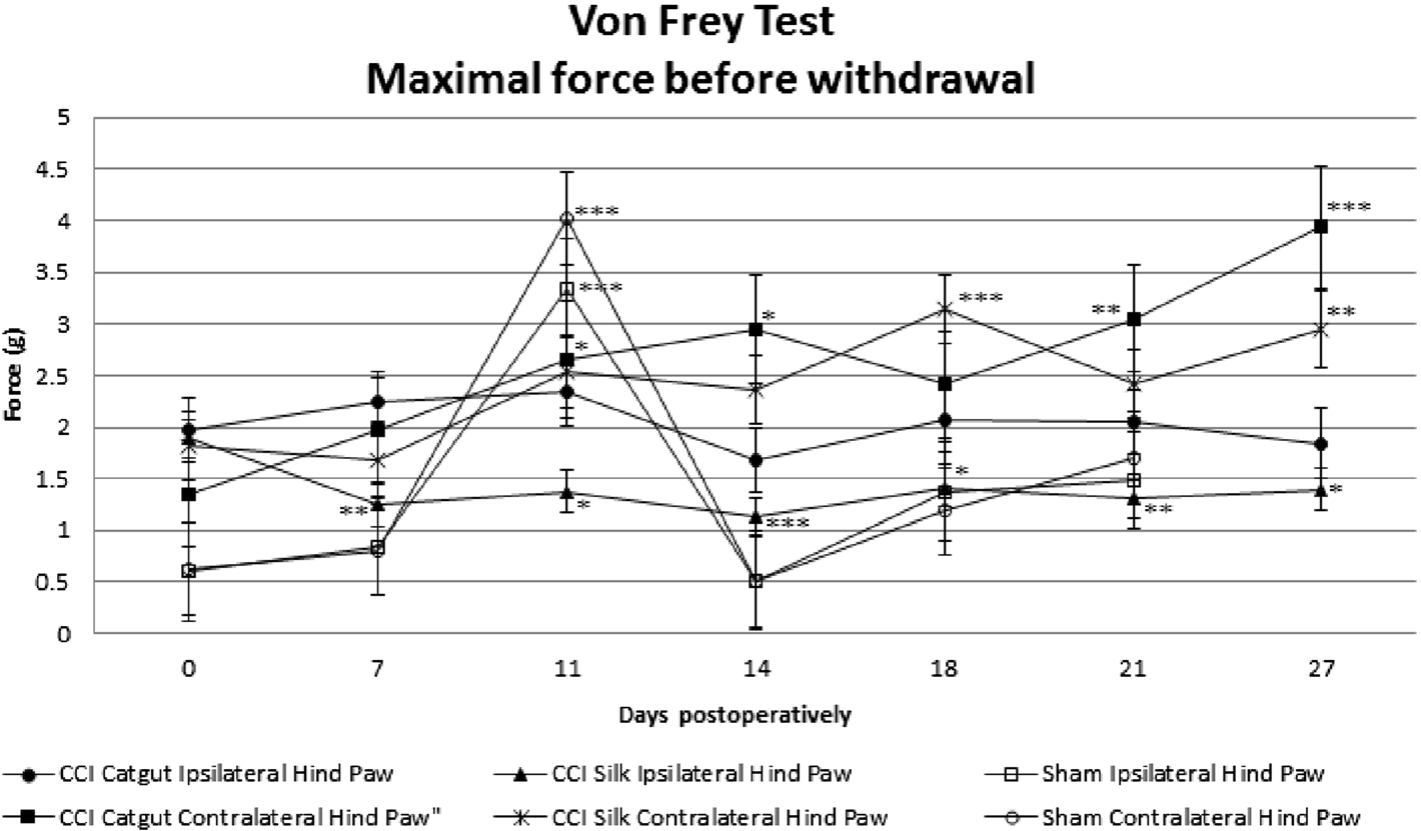
Mechanical allodynia measured in CCI mice and sham mice with the Von Frey test in maximum force before withdrawal ±SE preoperatively and postoperatively in both ipsilateral (left) and contralateral (right) hind paw. Statistics were done with linear mixed models in which: *p<0.05 as compared with preoperative measurement, **p<0.01 as compared with preoperative measurement, ***p<0.001 as compared with preoperative measurement. Considering the right hind paw the CCI mice, both catgut and silk ligatured, showed a significant increase in the maximal force before withdrawal. Considering the left hind paw no significant difference was found in the catgut ligatured mice. In the silk ligatured mice a significant decrease was found in all postoperative measurements compared with the preoperative measurements.

Mechanical allodynia was tested in the von Frey test (Figure 4). Sham mice showed overall a low force before withdrawal, but on day 11 sham mice showed a peak in maximal force before withdrawal. The right hind paw showed an increase in maximal force before withdrawal. The left hind paw showed a significant decrease in maximal force before withdrawal, in silk ligatured mice more than in catgut ligatured mice. In catgut ligatured mice an increase in maximal force before withdrawal was seen until day 11. On day 14 a drop was seen, and after that a small increase was seen. Postoperatively there were no significant differences compared with preoperatively. In the silk ligatured mice a significant decrease in maximal force before withdrawal was seen on day 7. From day 7 the maximal force before withdrawal remained constant. This indicates mice lift their hind paw at lower force following CCI. The maximal force before withdrawal was significantly different in the contralateral hind paw than the sham mice. Between the ipsilateral hind paw and the sham mice only differences were seen preoperatively, on day 7 and day 10. Silk ligatured mice showed more mechanical allodynia than catgut ligatured mice (p=0.023).

## Discussion

In this study we demonstrated that a chronic constrictive injury (CCI) model in mice can induce neuropathic pain behaviors comparable to neuropathic pain signs and symptoms in humans. Chronic constriction injury in mice seems to present significant quantitative changes proportional to external stimulation in thermal and chemical hyperalgesia, thermal allodynia and mechanical allodynia. Neuropathy was developed from day 7 postoperatively and in most animals neuropathy was still observed until day 21–27 days postoperatively.

We tested two ligature materials, silk and catgut. Silk seems to be preferable compared with catgut as ligature material in mice.

### Neuropathic pain behavior

After surgery abnormal movements were seen in all groups, which disappeared mostly after one week. No mice had to be excluded from the experiment due to extensive weight loss, disease or autotomy. In some mice a slight reddening of the plantar surface of the toes was seen, these mice were not excluded from the experiment. It could however indicate that the ligature of the sciatic nerve was too tight. According to cumulative reaction time, measured with the acetone test, thermal and chemical hyperalgesia were present from day 7 in CCI mice with both silk and catgut ligatures. Sham mice were barely responsive to acetone application, but CCI mice showed associated aversive behavior as licking of the affected paw, limping with the left paw and enlarged movements. The acetone test was only performed in the ipsilateral hind paw, so no comparison with the contralateral hind paw could be made. Also according to both cumulative reaction time and number of lifts, measured with the cold plate test, cold allodynia was present in CCI mice with silk and catgut ligatures from day 7, compared with the contralateral hind paw and the sham mice. And also the von Frey test showed neuropathy in CCI mice with silk catgut ligatures from day 7.

The CCI-model has its limitations. For example, to obtain validated results, environmental factors should be eliminated. Concerning the acetone test the duration of exposure to cold is dependent on the spread and the evaporation of acetone, because of the ambient temperature and the body temperature of the mouse (Allchorne et al. 2005; Tanimoto-Mori et al. 2008). Furthermore the landing of the acetone of the plantar surface of the left hind paw is technique-dependant, and causes differences in mice (Vissers & Meert 2005). Also the liquid itself may elicit a chemical, olfactory or mechanical stimulus that may, independent of the temperature, elicit a flexion reflex (Vissers & Meert 2005; Allchorne et al. 2005; Tanimoto-Mori et al. 2008). Concerning the cold plate testing, not all tests could be performed due to technical difficulties, especially in the catgut-ligatured mice. Cold allodynia in mice can mimic cold allodynia observed in patients (Toyama et al. 2014). Cold plate testing has high behavioral variability and is mainly used for neuropathy models. During cold plate testing we did found high baseline values in the cold plate tests with high variability. Habituation is a contributing factor in the gradual decline of our measurements and perhaps a longer period of acclimatization should be applied to research with cold plate testing in mice (Brenner et al. 2014). Concerning the von Frey testing, the bending forces applied by Von Frey filaments are significantly influenced by ambient humidity and slightly by temperature. Also washing and drying can significantly affect the bending forces (Andrews 1993). It is important the experimenter waits for the animal to hold its paw in the right position as weight bearing of the limb might be a confounding factor in determining von Frey withdrawal thresholds. Therefore also the increased weight of the CCI mice during the experiment could be a determent factor in the von Frey test (Kauppila et al. 1998). In further research it might be useful to use an electronic von Frey meter, because of the difference in increase of the forces.

### Silk versus catgut

In the acetone test, the cold plate test and the von Frey test a trend toward more neuropathic behavior was shown in mice with silk ligatures compared with catgut ligatures. Robinson et al. showed catgut ligation caused cold allodynia, chemical hyperalgesia and mechanical hyperalgesia for at least 56 days post-surgery following partial sciatic nerve ligation (PNL) in rat. Silk ligatures caused the same deficits, but several of these deficits diminished over time 21–28 post surgery. In contrary to the rat model, where catgut is mostly used presumably because it induces an inflammatory response, in mice, catgut does not seem to be as effective. Perhaps catgut in mice does not induce an inflammatory response where silk ligature material does (Robinson & Meert 2005). However this research just measured neuropathic pain for 27 days, sham even 21 days, so the long term effect of ligature material on neuropathy is not studied and inflammatory parameters have not been studied. More research is therefore needed to prove our hypothesis that silk is more effective than catgut in mice in a CCI model.

## Conclusion

In conclusion this study demonstrated that a murine chronic constriction injury model to study neuropathic pain behavior can be a valuable model for testing of neuropathic pain and observational studies. Because mice are genetically modifiable, chronic constriction injury research in mice could create important opportunities in for example the role of inflammatory receptors or channel pathology compared with other animal models for the further discovering and testing of the mechanisms of neuropathic pain and possible new treatment targets.
